# Determinants of sepsis knowledge: a representative survey of the elderly population in Germany

**DOI:** 10.1186/s13054-018-2208-5

**Published:** 2018-10-28

**Authors:** Sarah Eitze, Carolin Fleischmann-Struzek, Cornelia Betsch, Konrad Reinhart, the vaccination60+ study group, Cornelia Betsch, Constanze Rossmann, Mathias W Pletz, Horst C Vollmar, Antje Freytag, Ole Wichmann, Regina Hanke, Wolfgang Hanke, Dorothee Heinemeier, Philipp Schmid, Sarah Eitze, Winja Weber, Anne Reinhardt, Nora K Küpke, Christina Forstner, Carolin Fleischmann-Struek, Anna Mikolajetz, Josephine Römhild, Julia Neufeind, Thorsten Rieck, Kasia Suchecka, Konrad Reinhart

**Affiliations:** 1 Center for Empirical Research in Economics and Behavioral Sciences, Department of Social Media and Communication Sciences, University of Erfurt, Nordhaeuser Strasse 63, 99089 Erfurt, Germany; 2 Integrated Research and Treatment Center for Sepsis Control and Care (CSCC), Jena University Hospital, Jena, Germany; 3 Department of Anesthesiology and Intensive Care Medicine, Jena University Hospital, Jena, Germany

**Keywords:** Sepsis, Knowledge, Elderly, Healthcare education

## Abstract

**Background:**

Sepsis is a life-threatening medical emergency requiring early diagnosis and urgent treatment. Knowledge is crucial, especially in major risk groups such as the elderly. We therefore assessed sophisticated knowledge about sepsis in the German elderly population.

**Methods:**

A telephone survey was carried out with a representative sample of 701 Germans from 16 federal states and a separate cohort of 700 participants from Thuringia, all aged ≥ 60 years. Sepsis knowledge was assessed via a 10-item questionnaire. Sociodemographic data and health information sources were assessed to identify determinants of sepsis knowledge.

**Results:**

Of the participants, 88.6% had heard the term “sepsis” before; however, 50% of these failed to define sepsis correctly. Even if the knowledge of symptoms was moderately good, most participants could not correctly identify causes of sepsis and underestimated its incidence. Only a minority was aware that immunization may prevent sepsis. Regressions revealed that being younger, better educated and living in rural areas predicted higher levels of sepsis knowledge. Pharmacists were a relevant source of sepsis information.

**Conclusions:**

Despite overall awareness of sepsis, the understanding of its risk factors, symptoms and prevention is low in the elderly, with important implications for emergency and intensive care. We suggest further educational measures to improve early sepsis recognition and prevention through vaccination.

**Electronic supplementary material:**

The online version of this article (10.1186/s13054-018-2208-5) contains supplementary material, which is available to authorized users.

## Background

Sepsis is the primary cause of death from infection and a medical emergency requiring early recognition and treatment [1]. Sepsis is responsible the death of over 6 million people per year worldwide [2]. In 2013, approximately 280,000 hospital patients were diagnosed and treated for sepsis in Germany. Approximately 70,000 of these patients died [3]. Delays in treatment are associated with increased risk of mortality [4]. Knowledge about early sepsis symptoms and the urge to seek emergency medical treatment are crucial to initiate early treatment, especially in major risk groups like the elderly or persons with comorbidities. To date, studies reveal substantial gaps in sepsis knowledge and perceptions in various countries [5–7]. In 2009, an international survey including 6021 participants from Europe and the USA revealed that 88% of the participants had never heard the term sepsis before [5]. Compared to stroke and myocardial infarction, perception of sepsis severity was also low in a Korean survey [7]. Even high-risk groups are not aware that vaccination protects against sepsis [8]. National and international initiatives such as World Sepsis Day aim to increase awareness and knowledge about sepsis. Overall, a recent WHO resolution on sepsis [2, 9] demands “increased public awareness of the risk of progression to sepsis from infectious diseases through health education”.

In order to design optimal health education provisions, it is crucial to assess perceptions of elderly Germans as the major risk group for sepsis and identify the relevant knowledge gaps [10] and the determinants of knowledge. For this purpose, we conducted a representative telephone survey. Based on a literature review, we identified potentially important determinants of knowledge in two categories: sociodemographic variables [11] and health information sources [12, 13]. This study is part of a prospective, multimethod intervention study aiming to increase influenza and pneumococcal vaccination rates and knowledge about sepsis in individuals aged ≥ 60 years in a model region in Germany, the federal state of Thuringia [14]. In order to evaluate the intervention’s effect on sepsis knowledge, this study serves as a baseline prior to the intervention. Therefore, it is important to assess similarities and differences of the Thuringian elderly compared to the nationwide sample.

## Methods

### Study design

The cross-sectional surveys comprise 701 participants for the German sample and 700 participants for the Thuringian sample. Inclusion criteria were age (≥ 60 years), language (German) and cognitive ability to answer the questions. The sample size is selected to obtain similar sample sizes for pre and post measurement of the health campaign vaccination60+. As preregistered in the study protocol of vaccination60+ [14], representativeness concerning the population over 60 years of age was established through information from census data about age, gender, education and residence in urban/rural environments of the target group. A power analysis determined the sample size for inferential statistics (independent *t* test: power = 0.8, alpha = 0.05, effect size *d* = 0.2) and rounded to the nearest higher hundred [14].

A professional survey company (Institute for Applied Marketing and Communication Research, IMK) conducted the computer-assisted telephone interviews (CATI) based on random digital dialing. With this widely used survey method [15, 16], it is possible to draw a random sample of the population [17]. The surveys took place between October 28 and December 16, 2016. The company received the final questionnaire and influenced neither its design nor analysis of the data.

### Measures

In a 72-item questionnaire, sepsis knowledge was assessed in addition to other scales and dimensions for psychological research and media and communication sciences. Results relating to vaccine hesitancy and media use will be reported elsewhere. The verbatim measures and data are available online (https://osf.io/vcuyd/). Within this report, sepsis knowledge was the main dependent variable. As determinants we assessed sociodemographics and health information sources. The questionnaire was pretested for clarity and length with 30 participants.

#### Sepsis awareness

All participants were asked whether they had heard the term sepsis and the term blood poisoning before. If they had, they were given further knowledge questions (*n* = 688 in the nationwide sample and *n* = 700 in the Thuringian sample). Those who knew only blood poisoning but not sepsis received the explanation that sepsis is often called “blood poisoning”. For all single-item analysis, we coded answers as correct = 2, incorrect = 1 and “don’t know” = 0.

#### Sepsis knowledge

Recent literature [5, 7, 11, 18] and expert interviews informed the design of the knowledge scale (see Table 3). The knowledge items assessed whether participants were aware that sepsis is a severe disease and what the definition of sepsis is as well as which treatment and prevention options exist. Five items contained correct statements, four items wrong statements: correct answers were coded as 1; incorrect and “don’t know” answers, and missing data were coded as 0. The participants also answered questions about possible symptoms of sepsis (fever/pain, tachycardia, tachypnea, abrupt cognitive impairment, hypotension). The correct identification of all symptoms and correct rejection of nonsymptoms was coded as 1 point. The mean of correct answers represents the total knowledge score, ranging from 0 to 1.

#### Determinants of sepsis knowledge

We asked participants about their highest formal educational level (e.g., secondary school or high school) and classified it in terms of the International Standard Classification of Education (ISCED-97) into low, middle and high educational status [19]. No further vocational achievements were coded. As additional sociodemographic determinants, we collected occupational status, rural/urban residence, health insurance status, age and gender. Participants also identified their frequently used sources of health information: healthcare workers, such as doctors, therapists and caregivers; brochures; classical media such as magazines, newspapers, radio and television; the Internet; and pharmacists.

### Statistical analyses

SPSS 24.0 was used for all analyses. Data and syntax are available online (https://osf.io/vcuyd/). Because the distribution of sampling criteria deviated from the census data, the data were weighted accordingly and thus can be regarded as representative of age, gender, urban/rural residency and educational level [20]. The distribution of sepsis knowledge is presented for the weighted data set. Weighted and unweighted data on the item level can be drawn from Additional file 1. For multiple regression analyses we used unweighted data. Sociodemographic variables were entered in the first block. Afterward, sources of health information were eliminated in a stepwise manner to identify information sources relevant for sepsis knowledge (backward regression).

## Results

**Fig. 1:**
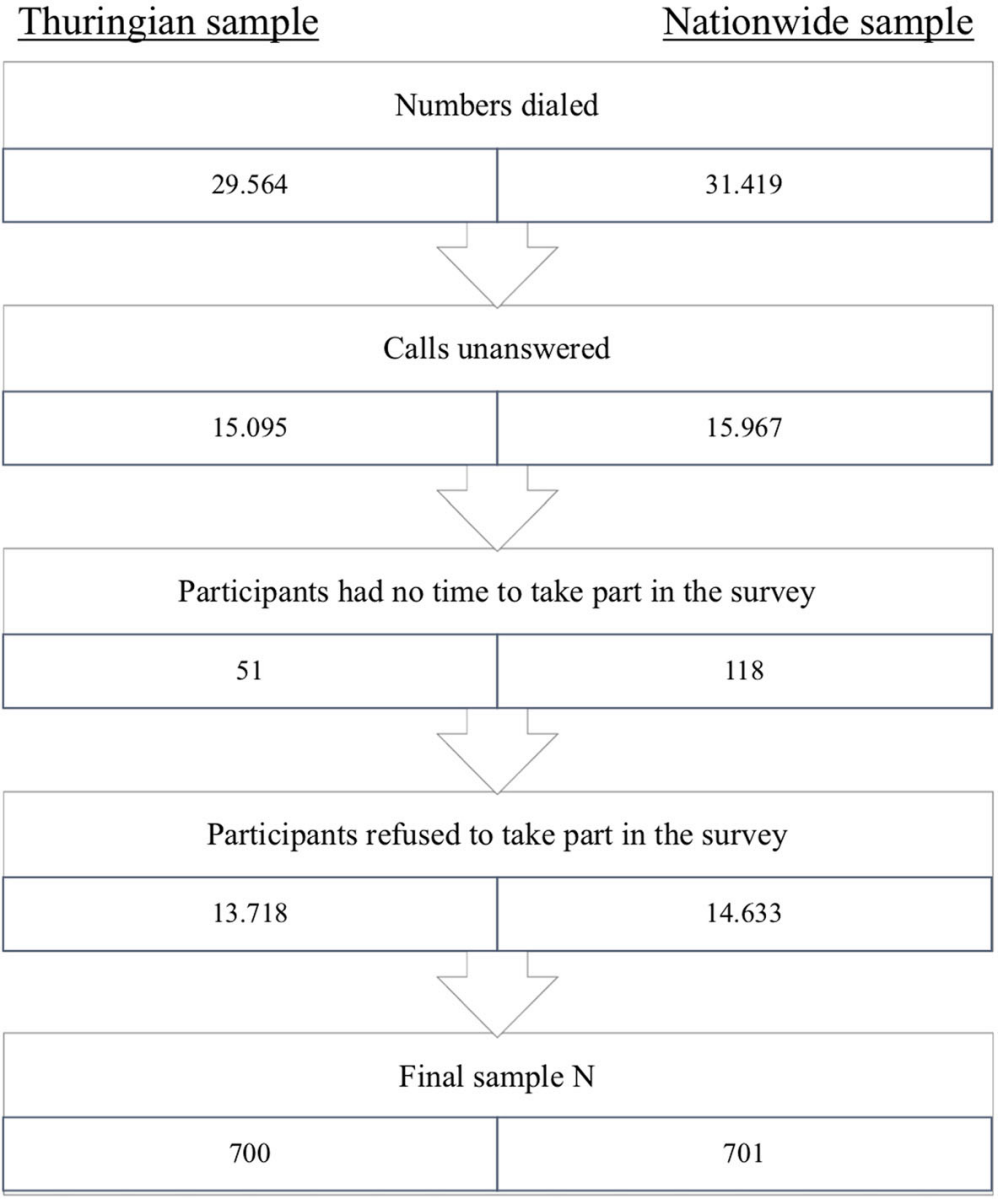
Flowchart of sample recruitment in Thuringian and nationwide surveys. Final response rates for samples were 2.2%

A total of 701 participants in the German sample and 700 participants in the Thuringian sample were interviewed. For the recruitment process, see Fig. 1. For demographics of the interviewees, see Table 1.

**Table 1:** Sociodemographic variables in Thuringian and nationwide samples (unweighted and weighted, according to Bethlehem et al. [20])

	Thuringian sample		Nationwide sample	
	Unweighted data	Weighted data	Unweighted data	Weighted data
Gender				
Male	253 (36.1)	308 (44.0)	318 (45.4)	313 (44.7)
Female	447 (63.9)	392 (56.0)	383 (54.6)	388 (55.3)
Age (years)				
60–69	310 (44.3)	326 (46.5)	318 (45.4)	308 (44.0)
70–79	273 (39.0)	266 (38.0)	268 (38.2)	278 (39.7)
80+	117 (16.7)	109 (15.5)	115 (16.4)	114 (16.3)
Education level				
Low	77 (11)	120 (17.2)	372 (53)	590 (85)
Intermediate	258 (36.9)	467 (66.7)	146 (20.8)	46 (6.6)
High	345 (49.3)	95 (13.5)	167 (23.8)	50 (7.2)
No data	20 (2.9)	18 (2.6)	16 (2.2)	14 (2)
Health insurance				
Statutory	654 (93.4)	670 (95.7)	519 (74)	569 (81.1)
Private	38 (5.4)	25 (3.6)	177 (25.2)	126 (18.0)
No data	8 (1.1)	5 (0.7)	5 (0.7)	6 (0.8)
Influenza vaccination	352 (50.3)	365 (52.1)	341 (48.6)	332 (47.4)
Pneumococci vaccination	190 (27.1)	168 (24.0)	139 (19.8)	141 (20.1)

Data presented as *N* (%). We coded education as low, medium and high, following the International Standard Classification of Education ISCED-97 classification. Vaccination status indicates the self-reported status for influenza in the 2016 season and pneumococcal vaccination during the previous 10 years

### Sepsis awareness and knowledge in the nationwide sample

Overall, the interviewees answered about one third of the knowledge items correctly (M = 0.3466, SD = 0.1815; for complete results of all items see Table 2). Of the participants, 88.6% had heard the term sepsis before the survey; 98.1% knew the term “blood poisoning” (total *n* = 688). Thirty-nine percent could not identify the correct definition of sepsis, namely that sepsis is an intensive immune reaction of the body to an infection. Of participants, 45.4% answered that sepsis is an intense allergic reaction and 29.7% that sepsis can be caused by multidrug-resistant killer germs in hospitals. Particularly noteworthy is that only 12.4% and 24% respectively knew that infections like influenza or pneumonia can cause sepsis. The analysis also identified several misbeliefs. The most prominent myth concerning sepsis was the belief that a red line to the heart comprises a crucial diagnostic sign of sepsis (58.1% thought this statement was correct, 19.9% were unsure). In terms of common sepsis symptoms, fever and chills, tachycardia and shortness of breath were identified correctly by 73%, 61.5% and 51.1%, respectively. Fewer interviewees were aware that abrupt cognitive impairment and hypotension are also common sepsis symptoms (32.1% and 19.3% respectively). The incidence of sepsis was underestimated compared to breast cancer. A total of 83.4% was aware that sepsis is an emergency to be immediately presented at the hospital, but interviewees underestimated the mortality of sepsis in comparison to heart attacks (“The mortality rate after heart attacks is higher than the mortality rate after sepsis”: 12.3% no, 50% yes, 34.9% unsure). Only 17.2% of participants knew that vaccinations can reduce the risk of sepsis.

**Table 2:** Awareness and sepsis knowledge: distribution of correct, incorrect and “don’t know” answers per item for the nationwide sample

	Weighted data		
	Yes	No	Unsure
Awareness items preceding the knowledge scale			
Have you ever heard of the term sepsis?	621 (88.6)	77 (11)	3 (0.4)
Is there a vaccination against sepsis?	121 (17.2)	368 (52.4)	133 (19)
Items integrated in Sepsis Knowledge Score (M = 0.3466, SD = 0.1815)			
With sepsis, you have to call the emergency services immediately	584 (83.4)	47 (6.8)	54 (7.8)
Sepsis is an intense allergic reaction	161 (22.9)	318 (45.4)	202 (28.8)
Sepsis is an intense immune response of the body	410 (58.5)	88 (12.5)	188 (26.8)
Sepsis is caused by multidrug-resistant superbugs in hospitals	208 (29.7)	275 (39.2)	201 (28.7)
Sepsis can be diagnosed by a red line infiltrating from a wound up to the heart	407 (58.1)	138 (19.7)	140 (19.9)
Mortality after heart attacks is higher than mortality after sepsis	350 (50)	86 (12.3)	245 (34.9)
There are more cases of breast cancer than cases of sepsis	273 (39)	107 (15.3)	302 (43)
Sepsis can be caused by lung inflammation	168 (24)	199 (28.4)	321 (45.8)
Sepsis can be caused by influenza	87 (12.4)	332 (47.4)	266 (38)
Sepsis Symptoms Score (M = 0.4718, SD = 0.2637)			
Are chills and fever symptoms of sepsis?	512 (73)	67(9.6)	106 (15.2)
Is disorientation a symptom of sepsis?	225 (32.1)	248 (35.4)	212 (30.2)
Is shortness of breath a symptom of sepsis?	358 (51.1)	155 (22.1)	172 (24.5)
Is a high heart rate a symptom of sepsis?	431 (61.5)	102 (14.5)	152 (21.7)
Is low blood pressure a symptom of sepsis?	135 (19.3)	298 (42.5)	252 (35.9)
Is diarrhea a symptom of sepsis?	135 (19.3)	370 (52.8)	180 (25.7)
Are skin rash and eczema symptoms of sepsis?	238 (34)	283 (40.4)	164 (23.4)

Data presented as *N* (%). Items in Sepsis Knowledge Score and in Sepsis Symptoms Score presented in randomized order

*SD* standard deviation

### Determinants of sepsis knowledge in the nationwide sample

Because some participants refused to give information about their insurance status, the following analysis includes 663 participants. A multiple linear regression analysis was used to develop a model for predicting sepsis knowledge from sociodemographic variables (first step) and sources of health information (second step) (*F*(7,655) = 7.598, *p* < 0.001; *R*^2^ = 0.075). An increase in knowledge was predicted by younger age (*β* = − 0.169, *p* < 0.001), higher education (*β* = 0.166, *p* < 0.001) and rural residence (*β* = − 0.079, *p* = 0.039). The only significant source of sepsis information was pharmacists (*β* = 0.128, *p* = 0.001). Brochures, physicians, nurses and media as health information sources did not contribute to the prediction and were therefore eliminated by the backward regression. Table 3 presents the results of the multiple regression with all predictors.

**Table 3:** Determinants of sepsis knowledge in a multiple regression (nationwide sample)

Model		*β*	*t*	Sig.	95% CI for *β*	
					LCI	UCI
1	(Constant term)		6.914	0.000	0.351	0.630
	Age	− 0.170	− 4.278	0.000	− 0.005	− 0.002
	Gender	0.070	1.840	0.066	− 0.002	0.047
	Education	0.160	4.006	0.000	0.020	0.058
	Job status	0.047	1.180	0.238	− 0.015	0.059
	Health insurance	− 0.050	− 1.267	0.205	− 0.052	0.011
	Residence	− 0.079	− 2.053	0.041	− 0.056	− 0.001
2	(Constant term)		6.458	0.000	0.318	0.597
	Age	− 0.169	− 4.287	0.000	− 0.005	− 0.002
	Gender	0.064	1.702	0.089	− 0.003	0.045
	Education	0.166	4.194	0.000	0.021	0.059
	Job status	0.047	1.186	0.236	− 0.014	0.058
	Health insurance	− 0.045	− 1.172	0.242	− 0.050	0.013
	Residence	− 0.079	− 2.070	0.039	− 0.056	− 0.001
	Source: Pharmacists	0.128	3.392	0.001	0.009	0.033

We coded age as continuous linear, education as linear (low, medium, high; following International Standard Classification of Education ISCED-97), job status as dichotomous (working = 1/retired = 2), health insurance as dichotomous (statutory = 1/ private = 2) and residence as population of hometown (over 10,000 inhabitants = 1/under 10,000 inhabitants = 2). For sources of health information, higher scores indicate more frequent use of respective sources (range 1–5)

*Sig*. significance value *p* for regression weight *β*, *CI* confidence interval, *UCI* upper end of confidence interval, *LCI* lower end of confidence interval

### Thuringian sample

**Fig. 2:**
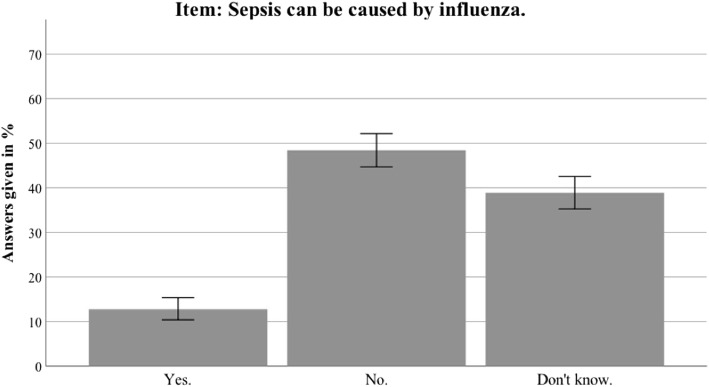
Nationwide distribution of sepsis knowledge about influenza as a possible origin. Bars indicate 95% confidence intervals

**Fig. 3:**
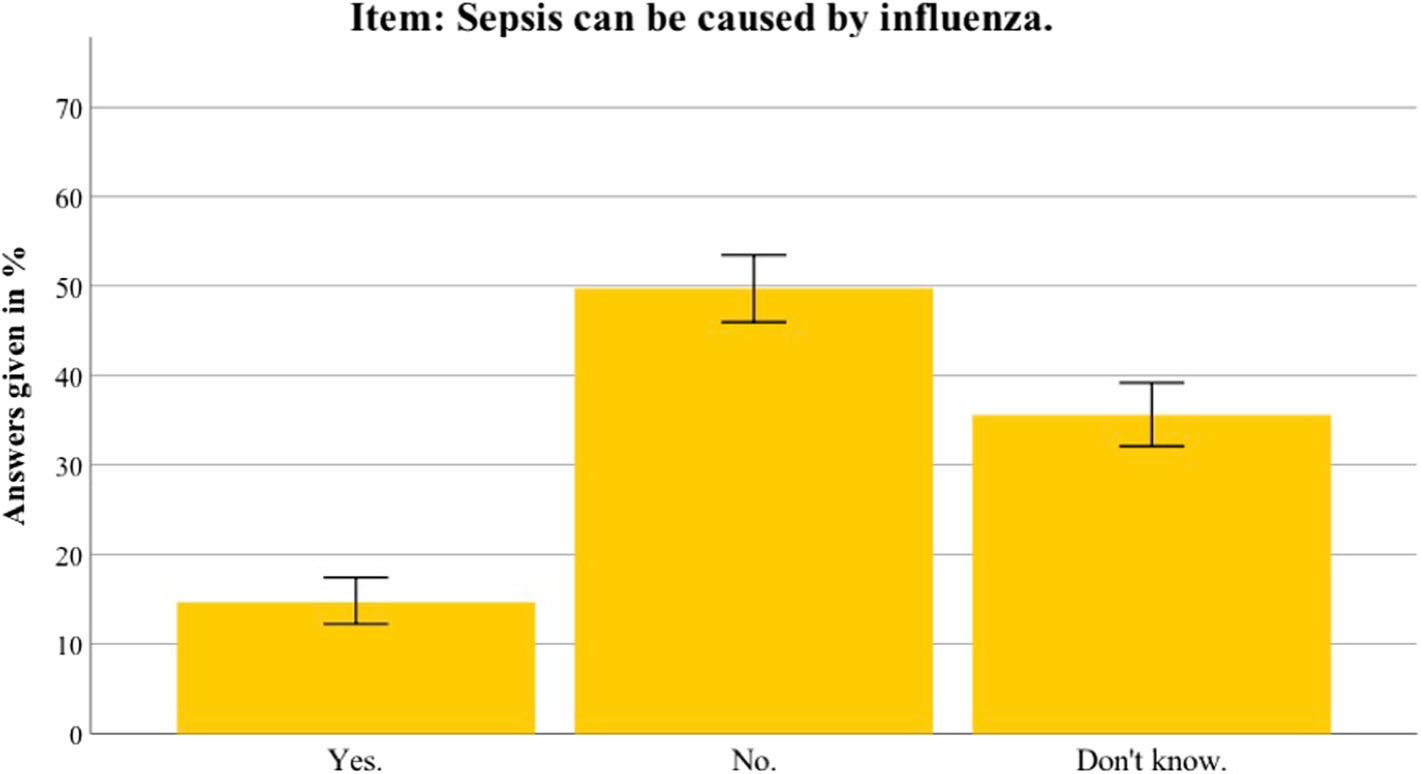
Thuringian distribution of sepsis knowledge about influenza as a possible origin. Bars indicate 95% confidence intervals

**Fig. 4:**
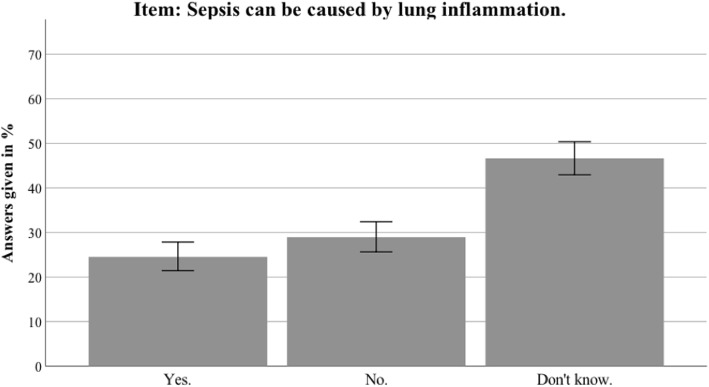
Nationwide distribution of sepsis knowledge about lung inflammation as a possible origin. Bars indicate 95% confidence intervals

**Fig. 5:**
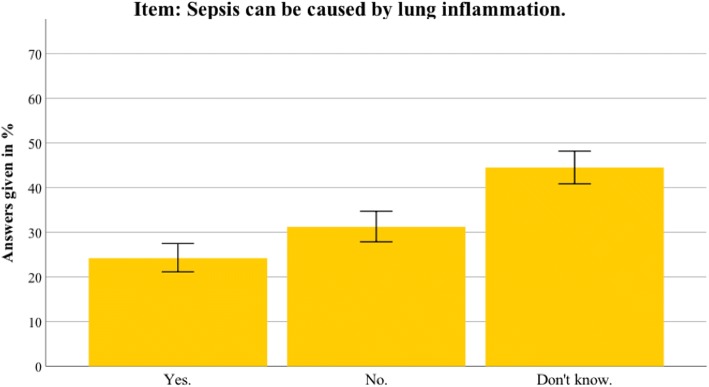
Thuringian distribution of sepsis knowledge about lung inflammation as a possible origin. Bars indicate 95% confidence intervals

**Fig. 6:**
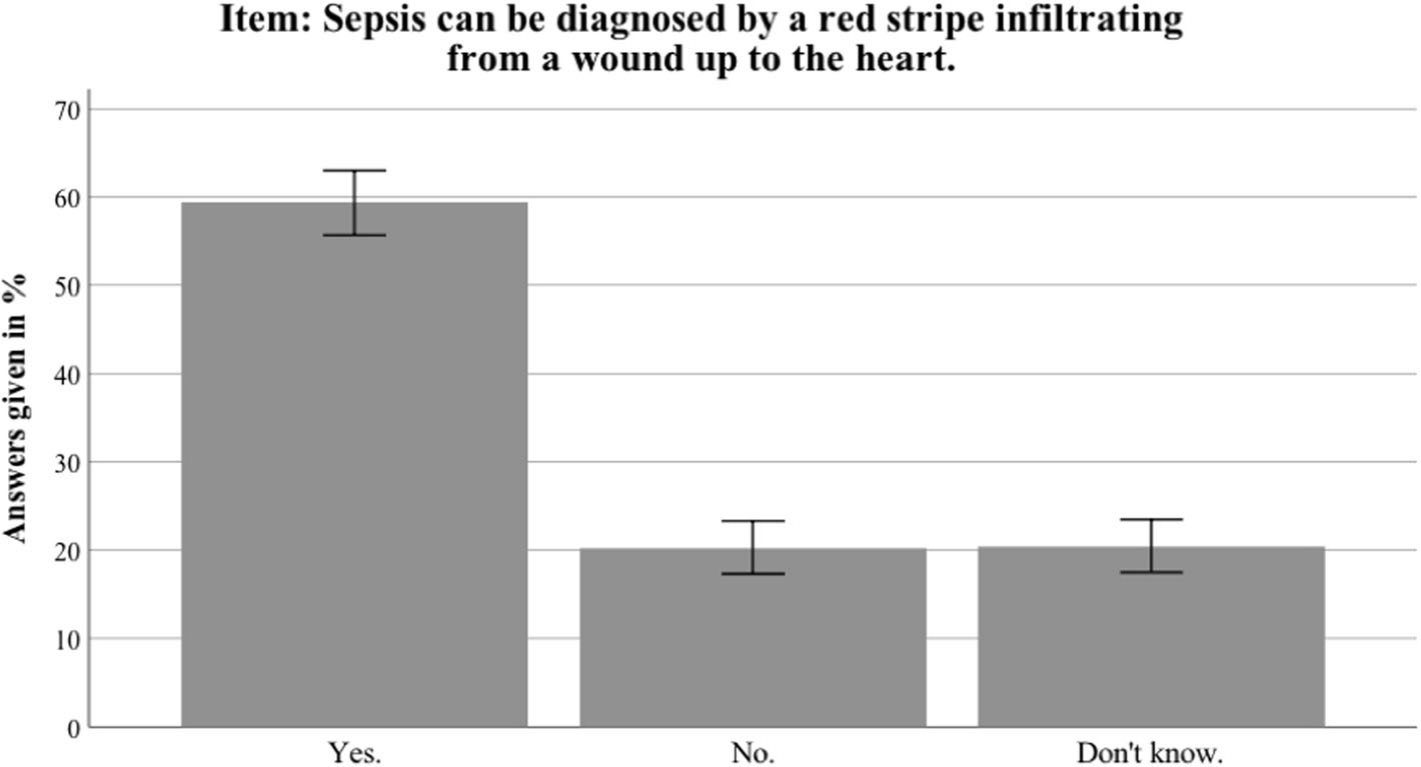
Nationwide distribution of most prominent myth about sepsis. Bars indicate 95% confidence intervals

**Fig. 7:**
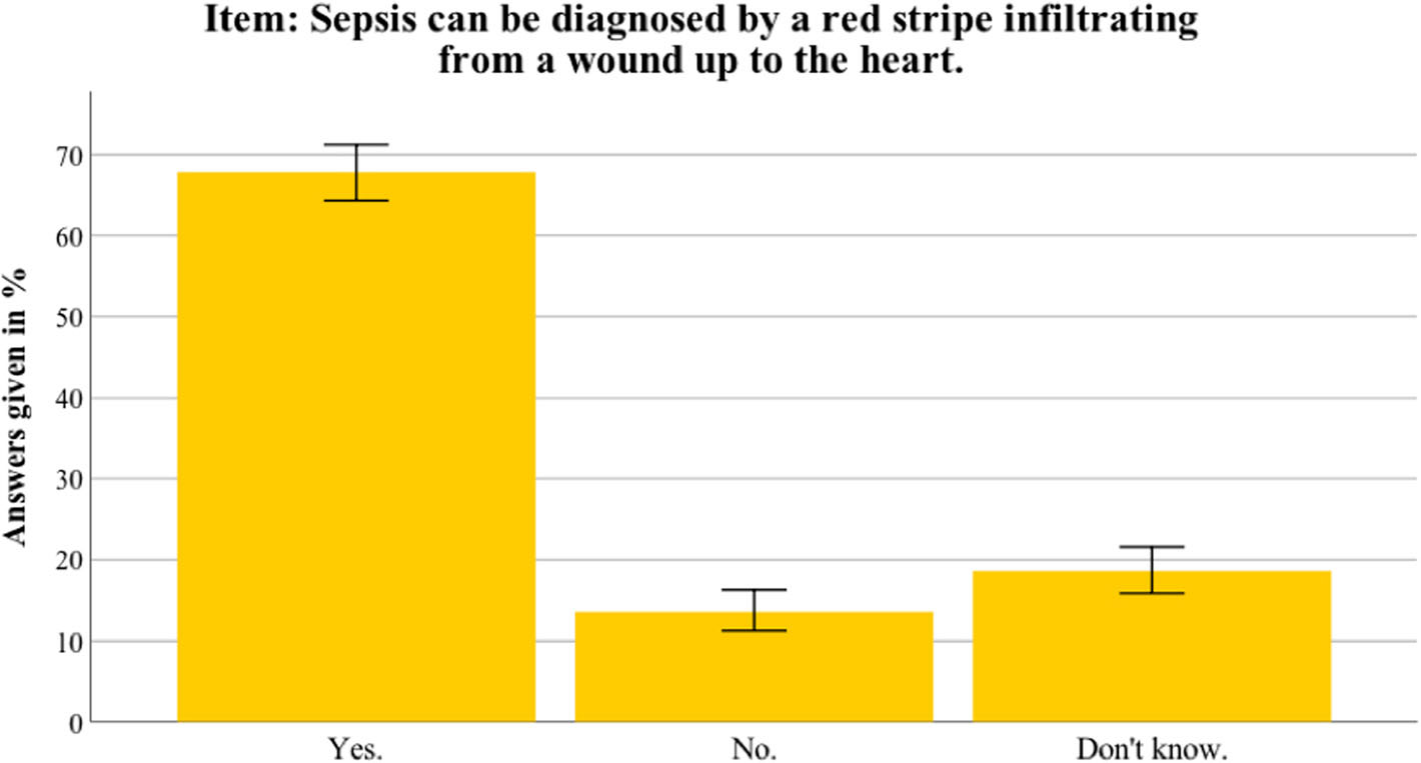
Thuringian distribution of most prominent myth about sepsis. Bars indicate 95% confidence intervals

**Fig. 8:**
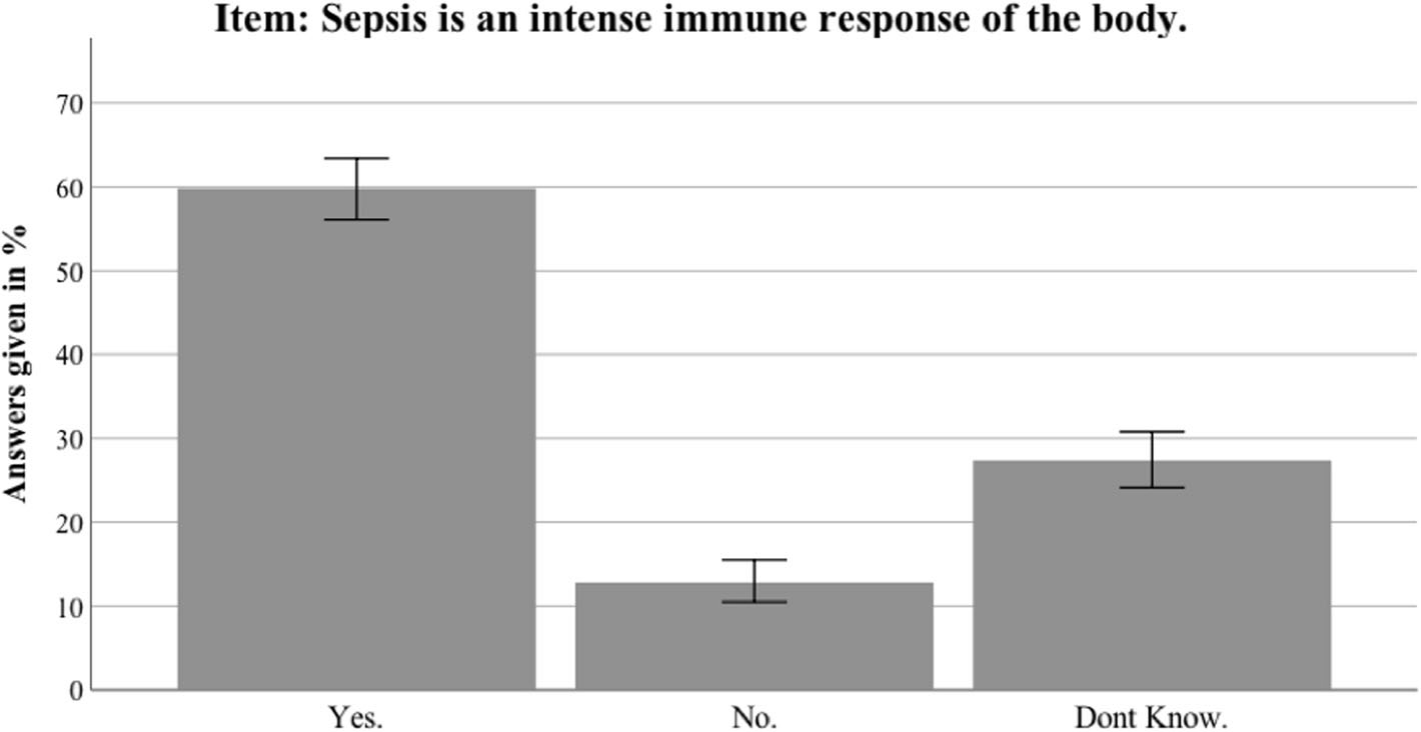
Nationwide distribution of correct definition of sepsis. Bars indicate 95% confidence intervals

**Fig. 9:**
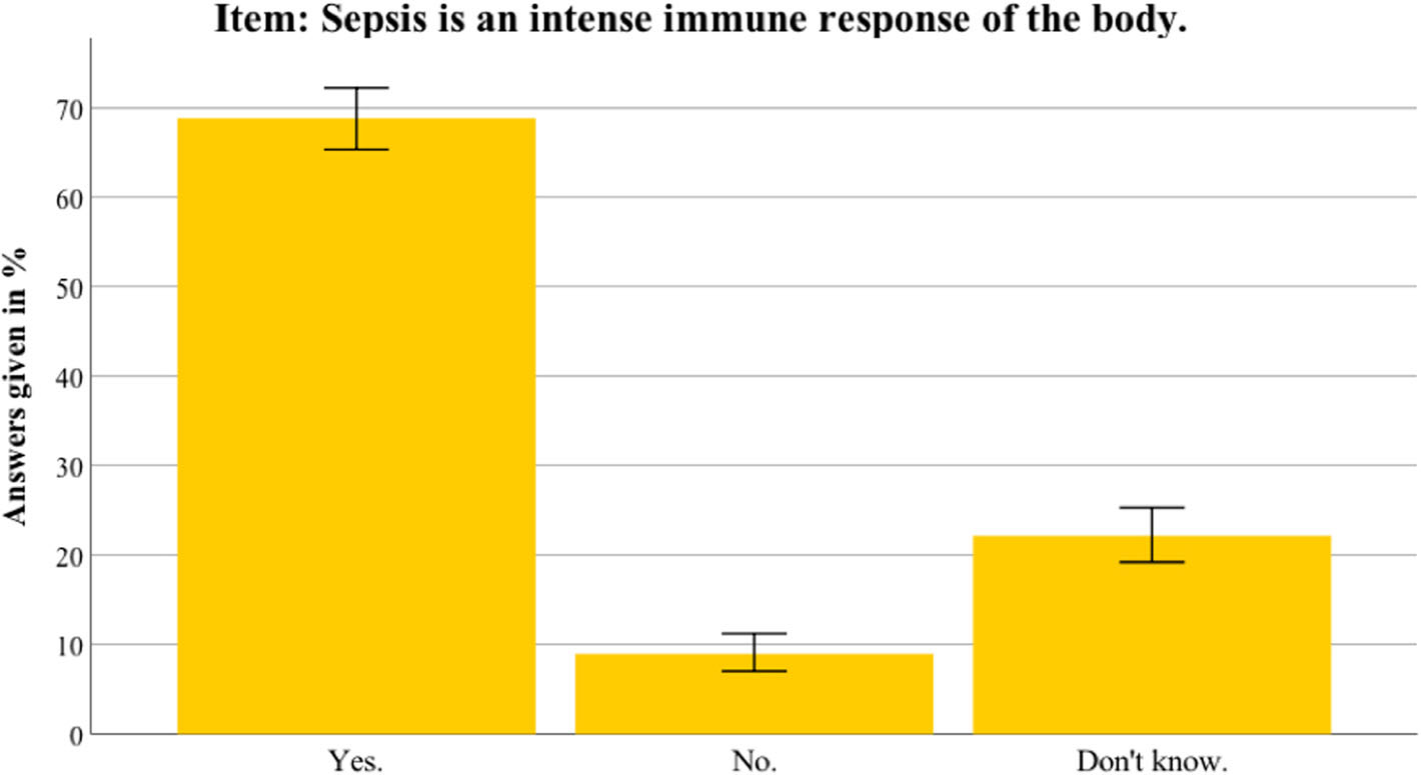
Thuringian distribution of correct definition of sepsis. Bars indicate 95% confidence intervals

**Fig. 10:**
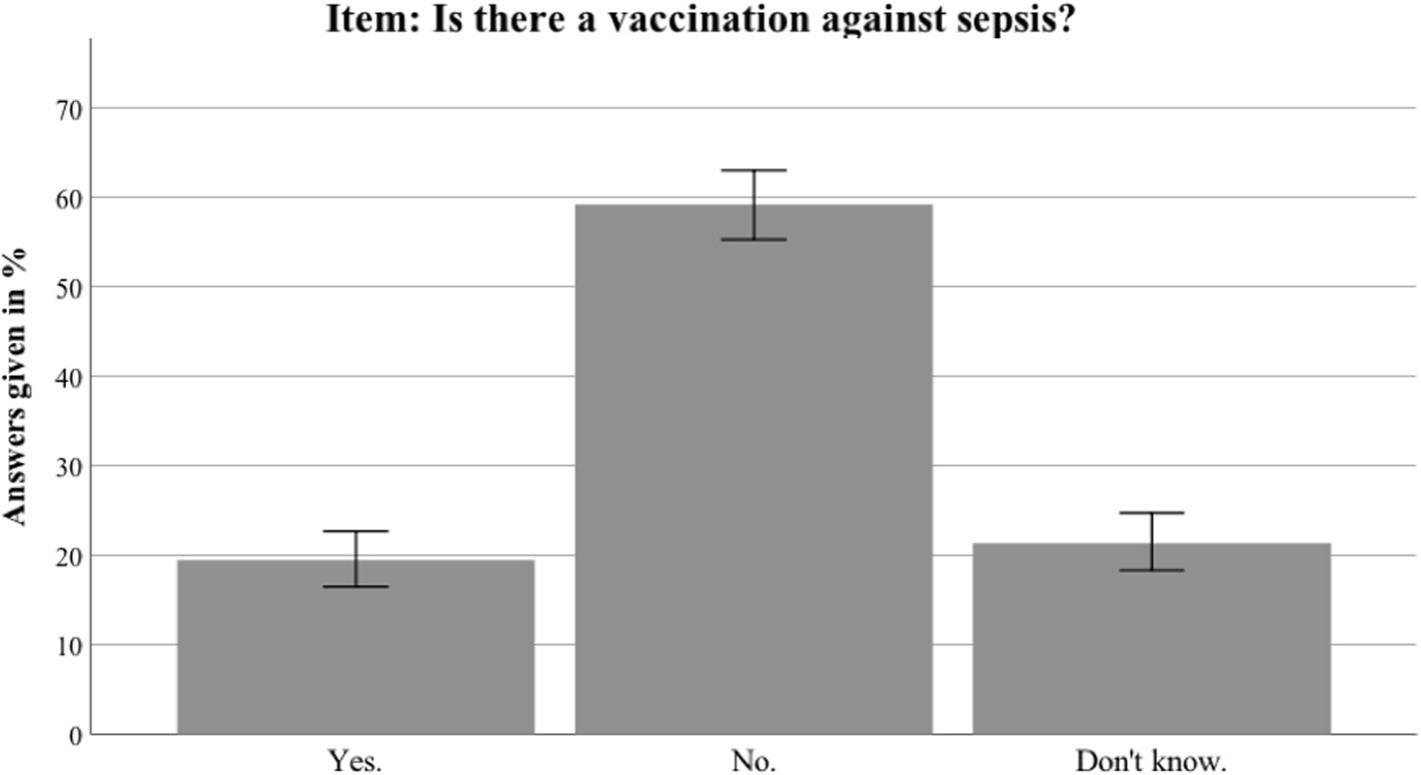
Nationwide distribution of awareness of sepsis prevention. Bars indicate 95% confidence intervals

**Fig. 11:**
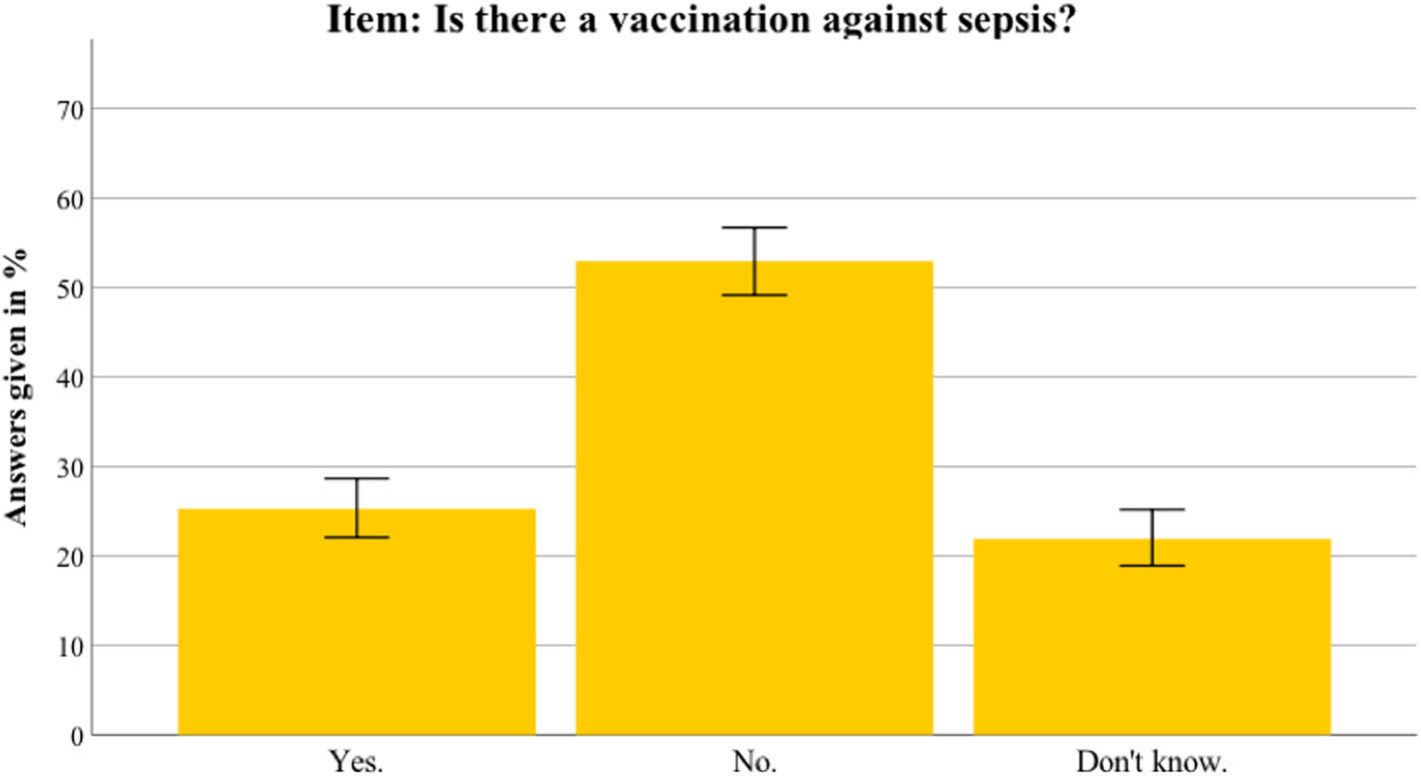
Thuringian distribution of awareness of sepsis prevention. Bars indicate 95% confidence intervals

Additional file 2 presents the detailed results for the Thuringian sample. In general, the results in Thuringia were similar to the nationwide data. This section therefore only highlights differences between the Thuringian sample and the German sample. Awareness of the term sepsis was higher in Thuringia than in the nationwide sample (94.5% vs 88.6%; *t*(1399) = 3.804, *p* < 0.001). Furthermore, the Thuringian sample identified sepsis correctly as an intense immune response (68.6% vs 58.5%; *t*(1381) = 3.107, *p* = 0.002). The most common sepsis myth (a red line under the skin being diagnostic for sepsis) was even more present in the Thuringian sample (67.7% vs 58.1%; *t*(1381) = 2.392, *p* = 0.017). For an overview of the most important sepsis knowledge items, see Figs. 2, 3, 4, 5, 6, 7, 8, 9, 10 and 11.

The same model was used to predict sepsis knowledge (*F*(8,669) = 11.73, *p* < 0.001; *R*^2^ = 0.12). Due to missing information about insurance status (*n* = 5) and educational level (*n* = 18), 680 participants were included in multiple regression analysis. In addition to age (*β* = 0.121, *p* = 0.002), Internet use (*β* = 0.245, *p* < 0.001) and classic media (*β* = 0.075, *p* = 0.041) were significant sources of sepsis knowledge. All other sources again had no significant partial effects in the full model (*p* > 0.05) and were eliminated by stepwise analysis. For an overview of the regression results see Additional file 2.

## Discussion

The survey presents the first comprehensive assessment of sepsis knowledge and its determinants in the German population aged 60 years and older, which comprises a major risk group for acquiring sepsis. In the federal state of Thuringia, recognition of the term “sepsis” was higher than in the nationwide sample and higher than in prior studies [5]. Nevertheless, profound comprehension as well as knowledge about the definition, symptoms and treatment of sepsis were low in both samples. The most prominent misbelief was that sepsis is characterized by a red line leading from a wound to the heart. Given that only a minority of participants knows that respiratory infections and influenza can lead to sepsis, it is not surprising that the interviewees were unaware that vaccinations may prevent sepsis. Beyond that, many important symptoms of sepsis were unknown, especially abrupt cognitive impairment and hypotension as early signals of sepsis. Even though the results revealed that this survey’s participants were aware that sepsis is an emergency, the majority of participants did not know the origins and signs of sepsis. They further underestimate the relative risk of dying from sepsis in comparison to other severe medical conditions. Thus, insufficient knowledge of sepsis symptoms and underestimation of disease severity may hamper timely presentation of patients in a healthcare facility, which can lead to a delay in medical treatment and increased mortality. Every delay in treatment is associated with an increase of mortality risk by 2% for the delay in antimicrobiological treatment and 1% for the delay in source control [4], therefore every hour in sepsis treatment is crucial. Younger age and higher education were determinants of increased sepsis knowledge. This suggests that providers and campaign designers should adapt the materials to older age groups and also present information for a less educated audience. Identifying important health information sources allows distributing education materials effectively, which is key to achieving the WHO resolution’s demand of increasing public awareness of sepsis [2].

The strength of this study is its representativeness regarding age, gender, education and living environment (urban/rural). The results contribute valuable knowledge regarding the design and content of education campaigns on sepsis [14]. Given the fact that more than 80% of sepsis cases are community acquired [21], education of the general public about seeking urgent medical treatment is critical in preventing avoidable deaths from sepsis. For other medical emergencies such as stroke or acute myocardial infarction, education campaigns have effectively increased awareness and reduced delay of initial treatment [22, 23]. National and international support by politicians, researchers and clinicians is crucial [9]. To achieve this goal, it is necessary to expand education of sepsis for healthcare professionals, who also have large gaps in knowledge about sepsis diagnosis and management [24, 25].

Sepsis awareness has considerably increased compared to results from the German Sepsis Foundation, which conducted surveys in 2013 and 2017 with 1014 participants, representative of the total population of Germany [26]. From 2013 to 2017, the survey found that within the population over 60 years of age the knowledge of the term sepsis increased by 9.7% to 64.7%. Apart from this lower estimation of sepsis awareness, other results are comparable. In this study, only a minority (20–30%) was aware of influenza and pneumonia as causes of sepsis and knew that vaccination can prevent sepsis. Similarly, several international studies record lower sepsis awareness [5–7]: 21% of interviewees in Sweden [6], 55% in the USA [27] and 66% in UK had heard of the term sepsis before (75% in the age group > 75 years) [UK Sepsis Trust 2017: YouGov/Sepsis UK Survey Results, unpublished]. The US and the UK studies found that sepsis awareness was higher in older individuals, a fact that may contribute to the significantly higher sepsis awareness in our study. Beyond that, potential increases in knowledge about sepsis are probably due to high efforts in educating about sepsis. Especially in Thuringia, a higher awareness can be expected through the local presence of and efforts made by the Head Office of the Global Sepsis Alliance and the offices of the German Sepsis Foundation and German Sepsis Aid. Other reasons may lie in the following limitations of our study.

In order to reach the final sample of 700 participants, about 30,000 numbers had to be dialed for each survey. Fifty percent of the calls were unanswered or mismatched quotes (younger than 60 years of age) and 46.5% refused to take part, so the overall response rate for both studies is 2.2%. This may indicate an underrepresentation of people with lower education and/or aversion to talk about health-related topics. The weighting corrects for this bias, but especially in the regressions we cannot correct for these biases.

A very high number of participants were aware that when sepsis is suspected, immediate contact with a healthcare facility is required. Due to randomization of the questionnaire items and the possibility that the participant had answered several questions about the severity of sepsis before this question, this number may be artificially high due to anchoring effects [28]. Further, possible influences of other confounding factors to sepsis knowledge (e.g., career in the health sector or individual experiences with sepsis) cannot be excluded and remain the subject of future research.

In conclusion, our survey has shown that in the elderly – one of the major risk groups for sepsis – knowledge about sepsis, its origin and its prevention is limited. This is also true for the fact that sepsis follows a unique and time-critical clinical course, which in the early stages is highly amenable to treatment through early diagnosis and timely and appropriate clinical management. For these reasons, educational and awareness campaigns for the public are urgently needed. The results of this survey may help to design such campaigns in terms of content – the role of vaccination in prevention and early warning symptoms. The results also help clinicians to form expectations about their patients’ level of knowledge. Such campaigns are important elements to reduce the huge human and health economic burden of sepsis.

## Conclusions

- Even though awareness is increasing for the term sepsis, definition and early symptoms are not commonly known in the elderly.
- Causes of sepsis are mostly misattributed to wound infection only.
- The majority of the elderly population is unaware that immunization may prevent sepsis.
- Age, education, residence and communication with pharmacists can partially explain differences in sepsis knowledge.
- Further awareness and educational campaigns for the public are badly needed to reduce the huge burden of sepsis.

## Additional files

Additional file 1: Differences for weighting factor of weighted and unweighted data in the surveys. **Table S1.1.** Nationwide sample. **Table S1.2.** Thuringian sample. (DOCX 22 kb)

Additional file 2: Results for analyses of Thuringian sample. **Table S2.1.** Regression analysis table for Thuringian sample. (DOCX 108 kb)
