# Supplementary material for: Determinants of sepsis knowledge: a representative survey of the elderly population in Germany
Source: Crit Care. 2018 Oct 28;22:273. doi: 10.1186/s13054-018-2208-5 (PMC6204268; doi:10.1186/s13054-018-2208-5)
Supplement: Supplementary file 1 — Differences for weighting factor of weighted and unweighted data in the surveys. Table S1.1. Nationwide sample. Table S1.2. Thuringian sample. (DOCX 22 kb) [file 13054_2018_2208_MOESM1_ESM.docx]

Additional File 1: Differences for weighted and unweighted data in the surveys

Table 1.1: Weighted and Unweighted distribution of Sepsis Knowledge in the German Sample

| **Awareness items preceding the knowledge score** | | N (%) unweighted | | | N (%) weighted | | |
| --- | --- | --- | --- | --- | --- | --- | --- |
|  |  | yes | no | unsure | yes | no | unsure |
| Have you ever heard of the term sepsis? | | 637 (90.9) | 60  (8.6) | 4  (0.6) | 621 (88.6) | 77  (11) | 3  (0.4) |
| Is there a vaccination against sepsis? | | 120 (17.1) | 399 (56.9) | 118 (16.8) | 121 (17.2) | 368 (52.4) | 133  (19) |
| **Items integrated in the Sepsis Knowledge Score** | | N (%) unweighted | | | N (%) weighted | | |
|  |  | yes | no | unsure | yes | no | unsure |
| With sepsis, you have to call the emergency services immediately. | | 604 (86.2) | 42  (6.0) | 44  (6.3) | 584 (83.4) | 47  (6.8) | 54  (7.8) |
| Sepsis is an intense allergic reaction. | | 153 (21.8) | 348 (49.6) | 183 (26.1) | 161 (22.9) | 318 (45.4) | 202 (28.8) |
| Sepsis is an intense immune response of the body. | | 443 (63.2) | 78 (11.1) | 164 (23.4) | 410 (58.5) | 88 (12.5) | 188 (26.8) |
| Sepsis is caused by multidrug-resistant superbugs in hospitals. | | 215 (30.7) | 292 (41.7) | 178 (25.4) | 208 (29.7) | 275 (39.2) | 201 (28.7) |
| Sepsis can be diagnosed by a red line infiltrating from a wound up to the heart. | | 408 (58.2) | 151 (21.5) | 129 (18.4) | 407 (58.1) | 138 (19.7) | 140 (19.9) |
| The mortality after heart attacks is higher than the mortality after sepsis. | | 369 (52.6) | 79 (11.3) | 238 (34) | 350 (50) | 86 (12.3) | 245 (34.9) |
| There are more cases of breast cancer than cases of sepsis. | | 274 (39.1) | 124 (17.7) | 285 (40.7) | 273 (39) | 107 (15.3) | 302 (43) |
| Sepsis can be caused by lung inflammation. | | 173 (24.7) | 214 (30.5) | 302 (43.1) | 168 (24) | 199 (28.4) | 321 (45.8) |
| Sepsis can be caused by influenza. | | 92 (13.1) | 339 (48.4) | 255 (36.4) | 87 (12.4) | 332 (47.4) | 266 (38) |
| **Sepsis Symptoms** | | N (%) unweighted | | | N (%) weighted | | |
|  |  | yes | no | unsure | yes | no | unsure |
|  | Are chills and fever symptoms of sepsis? | 532 (75.9) | 60  (8.6) | 95 (13.6) | 512 (73) | 67  (9.6) | 106 (15.2) |
|  | Is disorientation a symptom of sepsis? | 247 (35.2) | 240 (34.2) | 198 (28.2) | 225 (32.1) | 248 (35.4) | 212 (30.2) |
|  | Is shortness of breath a symptom of sepsis? | 348 (49.6) | 164 (23.4) | 175 (25.0) | 358 (51.1) | 155 (22.1) | 172 (24.5) |
|  | Is a high heart-rate a symptom of sepsis? | 451 (64.3) | 95 (13.6) | 141 (20.1) | 431 (61.5) | 102 (14.5) | 152 (21.7) |
|  | Is low blood pressure a symptom of sepsis? | 144 (20.5) | 311 (44.4) | 232 (33.1) | 135 (19.3) | 298 (42.5) | 252 (35.9) |
|  | Is diarrhea a symptom of sepsis? | 135 (19.3) | 374 (53.4) | 178 (25.4) | 135 (19.3) | 370 (52.8) | 180 (25.7) |
|  | Are skin rash and eczema symptoms of sepsis? | 257 (36.7) | 274 (39.1) | 154 (22) | 238 (34) | 283 (40.4) | 164 (23.4) |

Table 1.2: Weighted and Unweighted distribution of sepsis knowledge in the Thuringian Sample

| **Awareness items preceding the knowledge score** | | N (%) unweighted | | | N (%) weighted | | | |
| --- | --- | --- | --- | --- | --- | --- | --- | --- |
|  |  | yes | no | unsure | yes | no | | unsure |
| Have you ever heard of the term sepsis? | | 668 (95.4) | 30  (4.3) | 2  (0.3) | 664 (94.8) | 32  (4.6) | | 4  (0.6) |
| Is there a vaccination against sepsis? | | 165 (23.6) | 381 (54.4) | 121 (17.3) | 167 (23.9) | 351 (50.2) | | 145 (20.7) |
| **Items integrated in the Sepsis Knowledge Score** | | N (%) unweighted | | | N (%) weighted | | | |
|  |  | yes | no | unsure | yes | no | | unsure |
| With sepsis, you have to call the emergency services immediately. | | 623 (89.0) | 25  (3.6) | 49  (7.0) | 620 (88.5) | 24  (3.4) | | 50  (7.1) |
| Sepsis is an intense allergic reaction. | | 202 (28.9) | 311 (44.4) | 186 (26.6) | 215 (30.7) | 289 (41.3) | | 196 (28.0) |
| Sepsis is an intense immune response of the body. | | 480 (68.6) | 63  (9.0) | 153 (21.9) | 481 (68.6) | 62  (8.9) | | 155 (22.1) |
| Sepsis is caused by multidrug-resistant superbugs in hospitals. | | 289 (41.3) | 245 (35.0) | 163 (23.3) | 283 (40.5) | 246 (35.1) | | 169 (24.2) |
| Sepsis can be diagnosed by a red line infiltrating from a wound up to the heart. | | 438 (62.6) | 134 (19.1) | 126 (18.0) | 447 (67.7) | 95 (13.6) | | 130 (18.5) |
| The mortality after heart attacks is higher than the mortality after sepsis. | | 334 (47.7) | 94 (13.4) | 267 (38.1) | 349 (49.9) | 101 (14.4) | | 239 (34.2) |
| There are more cases of breast cancer than cases of sepsis. | | 248 (35.4) | 129 (18.4) | 317 (45.3) | 246 (35.2) | 128 (18.3) | | 318 (45.5) |
| Sepsis can be caused by lung inflammation. | | 180 (25.7) | 206 (29.4) | 311 (44.4) | 169 (24.2) | 218 (31.2) | | 311 (44.4) |
| Sepsis can be caused by influenza. | | 103 (14.7) | 339 (48.4) | 253 (36.1) | 102 (14.6) | 345 (49.3) | | 247 (35.3) |
| **Sepsis Symptoms** | | N (%) unweighted | | | N (%) weighted | | | |
|  |  | yes | no | unsure | yes | no | | unsure |
|  | Are chills and fever symptoms of sepsis? | 529 (75.6) | 68 (9.7) | 96 (13.7) | 503 (71.9) | 80 (11.4) | | 109 (15.5) |
|  | Is disorientation a symptom of sepsis? | 264 (37.7) | 244 (34.9) | 185 (26.4) | 238 (34) | 254 (36.2) | | 200 (28.6) |
|  | Is shortness of breath a symptom of sepsis? | 348 (49.7) | 177 (25.3) | 168 (24) | 331 (47.3) | | 184 (26.3) | 176 (25.2) |
|  | Is a high heart-rate a symptom of sepsis? | 443 (63.3) | 111 (15.9) | 138 (19.7) | 421 (60.1) | | 117 (16.8) | 151 (21.6) |
|  | Is low blood pressure a symptom of sepsis? | 150 (21.4) | 312 (44.6) | 230 (32.9) | 161 (23.1) | | 315 (44.9) | 211 (30.2) |
|  | Is diarrhea a symptom of sepsis? | 132 (18.9) | 376 (53.7) | 184 (26.3) | 126 (18) | | 369 (52.8) | 196 (28) |
|  | Are skin rash and eczema symptoms of sepsis? | 277 (39.6) | 235 (33.6) | 181 (25.9) | 252 (36) | | 245 (35.1) | 194 (27.7) |
