# Supplementary material for: Determinants of sepsis knowledge: a representative survey of the elderly population in Germany
Source: Crit Care. 2018 Oct 28;22:273. doi: 10.1186/s13054-018-2208-5 (PMC6204268; doi:10.1186/s13054-018-2208-5)
Supplement: Supplementary file 2 — Results for analyses of Thuringian sample. Table S2.1. Regression analysis table for Thuringian sample. (DOCX 108 kb) [file 13054_2018_2208_MOESM2_ESM.docx]

Additional Files 2: Results for the Analyses of the Thuringian sample

*Table 2.1*: Determinants of sepsis knowledge as regression weights for the Thuringian sample

| Model | | β | T | Sig. | 95.0% CI for β | |
| --- | --- | --- | --- | --- | --- | --- |
|  |  |  |  |  | LCI | UCI |
| 1 | (Constant Term) |  | 6.849 | .000 | .397 | .716 |
|  | Age | -.168 | -4.163 | .000 | -.005 | -.002 |
|  | Gender | -.102 | -2.666 | .008 | -.056 | -.009 |
|  | Education | .086 | 2.209 | .028 | .003 | .046 |
|  | Job Status | .023 | .584 | .559 | -.025 | .047 |
|  | Health insurance | -.052 | -1.361 | .174 | -.105 | .019 |
|  | Residence | .064 | 1.692 | .091 | -.004 | .050 |
| 2 | (Constant Term) |  | 5.132 | .000 | .258 | .578 |
|  | Age | -.116 | -2.909 | .004 | -.004 | -.001 |
|  | Gender | -.065 | -1.721 | .086 | -.044 | .003 |
|  | Education | .061 | 1.624 | .105 | -.004 | .038 |
|  | Job Status | .037 | .965 | .335 | -.018 | .052 |
|  | Health insurance | -.074 | -1.996 | .046 | -.122 | -.001 |
|  | Residence | .065 | 1.760 | .079 | -.003 | .049 |
|  | Source: Internet | .256 | 6.638 | .000 | .024 | .044 |
| 3 | (Constant Term) |  | 4.762 | .000 | .230 | .554 |
|  | Age | -.121 | -3.045 | .002 | -.004 | -.001 |
|  | Gender | -.071 | -1.887 | .060 | -.046 | .001 |
|  | Education | .067 | 1.767 | .078 | -.002 | .040 |
|  | Job Status | .040 | 1.023 | .307 | -.017 | .053 |
|  | Health insurance | -.073 | -1.961 | .050 | -.121 | .000 |
|  | Residence | .070 | 1.909 | .057 | -.001 | .051 |
|  | Source: Internet | .245 | 6.319 | .000 | .022 | .043 |
|  | Source: Classic Media | .075 | 2.043 | .041 | .000 | .020 |

*Note:* We coded age as a continuous variable, gender as male =1/female=2, education as linear (low, medium, high, following ISCED97), job status as dichotomous (working=1/retired=2), residence as population of hometown over=1/under 10.000=2 inhabitants. For the sources of health information higher scores indicate more frequent use of the respective sources (range 1-5).
